# Phytochemical Profiling, Bioactivity, and Insecticidal Effectiveness of *Mammea americana* L. Leaf Extracts Against *Ferrisia* sp.

**DOI:** 10.3390/plants14010021

**Published:** 2024-12-25

**Authors:** Mike Vázquez-Torres, Nilka Rivera-Portalatín, Irma Cabrera-Asencio

**Affiliations:** 1 Department of Chemistry, University of Puerto Rico, Mayaguez Campus, Mayaguez, PR 00681, USA; nilka.rivera1@upr.edu; 2 Laboratory of Entomology, Juana Díaz Agricultural Experiment Station, Department of Agro-Environmental Sciences, University of Puerto Rico, Mayaguez Campus, Mayaguez, PR 00681, USA; irma.cabreraasencio@upr.edu

**Keywords:** biological activity, mealybugs, phytochemistry, plant extracts, secondary metabolites

## Abstract

Plant botanical extracts are recognized for being a source of biologically active phytochemicals that potentially have diverse applications. The phytochemical composition, potential cytotoxicity, and insecticidal effectiveness of three leaf extracts from the folkloric medicinal plant *Mammea americana* L. (Calophyllaceae) were investigated. Micro-Soxhlet extraction with chloroform, dichloromethane, and methanol was used, and key phytochemicals were identified via Gas Chromatography-Mass Spectrometry (GC-MS). The extracts were mainly composed of sesquiterpenes, carboxylic acids, coumarins, esters, diterpenes, and other bioactive compounds. Potential cytotoxicity was assessed using brine shrimp lethality tests, where all extracts displayed high toxicity to *Artemia salina*. The dichloromethane extract (MAD) had the lowest LC_50_ value (8.39 μg/mL), followed by methanol extract (MAM, 11.66 μg/mL) and chloroform extract (MAC, 12.67 μg/mL). Insecticidal activity was tested against *Ferrisia* sp. (Hemiptera:Pseudococcidae), demonstrating the highest efficacy with the methanolic extract (LC_50_ = 5.90 mg/mL after 48 h). These findings provide a basis for further research into the bioactive components of *Mammea americana* leaves, particularly their antibacterial, anti-inflammatory, and anticancer properties. It also highlights the potential of *Mammea americana* L. leaf extracts as botanical insecticides due to their high bioactivity against agricultural pests of economic significance. This is the first study that evaluates the insecticidal activity of *Mammea americana* leaf extracts against *Ferrisia* sp. insects, offering valuable insights into using plant-based natural products in pest control.

## 1. Introduction

Natural products, such as plant extracts, are recognized for possessing many biological activities and/or medicinal properties related to the presence of a diverse mixture of chemical organic compounds or phytochemicals [1,2]. Phytochemicals or plant secondary metabolites, such as phenolics, flavonoids, quinones, tannins, alkaloids, saponins, and sterols, among others, play a key role in protecting plants or regulating their metabolism [3]. They can be isolated through volatilization without decomposition by physical methods from various parts of a plant, including leaves, flowers, seeds, bark, stem roots, and shrubs [1]. Soxhlet extraction is a conventional method used to extract bioactive chemical compounds from plant material, in which a wide range of organic solvents can be used to isolate the extractable compounds [4]. The chemical constituents of the volatile and semi-volatile samples obtained by Soxhlet extraction are excellent candidates for chemical analysis by Gas Chromatography coupled to Mass Spectrometry (GC-MS) since the mass selective detector provides the spectrum library database that allows the identification of unknown mixtures of compounds based on the defragmentation pattern spectra and their mass to charge (*m*/*z*) ratio [5].

*Mammea americana* L., commonly known as mammee, mammee apple, or mamey in Puerto Rico, is a wild evergreen tree of the family *Calophyllaceae*, formerly placed in the family *Guttiferae*, commonly found in deep, rich, well-drained soil [6,7,8]. Native to the West Indies and northern South America, it reaches 18 to 21 m in height and has glossy, dark green, broadly elliptic leaves of 8 to 20 cm long grouped in ascending branches and white fragrant flowers of 4 to 6 petals with orange stamens or pistils [7,8]. It is well known for its nearly round, irregular shape and light brown to grayish-brown edible fruit of 6 to 20 cm of fragrant, appetizing ripe flesh [8]. Several studies have reported the medicinal and toxic properties of various parts of this folkloric medicinal plant, including the bark, leaves, flowers, fruits, and seeds [7,8,9]. In a study of Mammee apple fruit, Janakiram et al. (2008) found that it was mainly composed of β-ionone, a compound that can exert anticarcinogenic and antitumor activities [10], while Frame et al. in 1998, concluded that *Mammea americana* L. (Calophyllaceae) yielded the strongest bactericidal inhibitory effect comparable to streptomycin [11]. Also, many isoprenylated coumarins that have exhibited significant cytotoxic activities against human colon cancer cell lines were identified by Yang et al. in 2005 in seed extracts from this plant [12]. The unripe skin of the fruit and the seeds have proved to be very toxic as a contact poison against mosquitoes and their larvae, while the gum from the bark melted with fat has been effective in combating chiggers ridding animals of fleas and ticks [7]. In the past, there was a practice of wrapping Mamey leaves around young tomato plants in Puerto Rico to protect them from mole crickets and cutworms [7]. In 1996, Gallo et al. reexamined the insecticidal effectiveness of *Mammea americana* L. (Calophyllaceae) in the search for renewable sources of botanical insecticides and reported that the crude hexane extracts from the leaves and seeds have insecticidal effect against *Diabrotica virgifera virgifera* and *Trichoplusia ni* larvae [13].

*Ferrisia* is a genus of insects from the order Hemiptera and family Pseudococcidae, which includes *Ferrisia virgata* and *Ferrisia dasylirii* (Cockerell), among others [14]. Both insects grow and live together in colonies, making it impossible to distinguish them by only simple superficial features, requiring to be slide-mounted [15]. These striped mealybugs are pests of several agricultural crops, including fruits, vegetables, and ornamentals, that they affect by sap-feeding, producing the honeydew that is a medium for the growth of the sooty mold impairing photosynthesis with their dark films in the plant tissues [14]. This problem has achieved economic significance, with an annual loss of millions in agriculture, making necessary their management by chemical control with pesticide sprays or systematic insecticides that can negatively impact the ecosystem and human health [16].

Plant botanical extracts can be alternatives to synthetic chemicals, being natural derivatives, since they are biodegradable, do not damage the environment, and are unlikely to cause resistance over time due to their phytochemical diversity and natural origin [3,17]. Focused on the need to find safer alternatives for agricultural pest control of mealybugs and having past scientific evidence of the effectiveness showed by *Mammea* leaves and seed extracts against other insects of agricultural interest, our principal goal was to investigate if the phytochemical mixture from *Mammea* leaf extracts also results in an effective botanical insecticide against *Ferrisia* sp. mealybugs. The efficacy of plant crude extracts against pests is important, along with the knowledge of the chemistry of pesticidal activity and bioactive components [18].

This study aimed to determine the phytochemical composition, the bioactivity and/or potential toxicity, and the insecticidal activity against *Ferrisia* sp. mealybugs of three different leaf extracts derived from *Mammea americana* L. (Calophyllaceae). We applied traditional Soxhlet extraction at the microscale to reduce the extensive use of solvents and optimize extraction processing periods [4]. Using non-polar to polar extraction solvents, we sought to improve the extraction of plant secondary metabolites for their subsequent chemical analysis. Volatile and semi-volatile components of the leaf extracts were separated and analyzed by Gas Chromatography-Mass Spectrometry (GC-MS) to characterize their phytochemical composition. The biological activity of the leaf extracts was assessed through the brine shrimp lethality test (BSLT) with *Artemia salina* [19]. First described by Meyer et al. 1982, it is still considered the most convenient, reliable, and inexpensive general bioassay method for frontline screening, predictive of cytotoxicity and pesticidal activity of natural products in environmental toxicity testing due to the versatility of *Artemia* as a model organism in stress response studies [19,20]. The insecticidal effectiveness of the leaf extracts was evaluated through contact toxicity bioassays against *Ferrisia* sp., which includes both striped mealybugs, *F. virgata,* and *F. dasylirii*. Currently, to our knowledge, this is the first study in which leaf extracts from *Mammea americana* are tested for their insecticidal activity against these pests of agricultural economic significance, providing valuable insights on the effectiveness of plant-derived natural products in crop protection and as a greener solution for agricultural pest control.

## 2. Results and Discussion

### 2.1. Extraction Yield with Different Solvents for Mammea americana L. Leaf Extracts

Micro-Soxhlet extraction of the deep dark greenish extract of *Mammea americana* L. (Calophyllaceae) fresh leaves yielded 40 ± 7% (*v*/*w*) with the non-polar solvent chloroform, 24.8 ± 0.6% (*v*/*w*) with the medium-polar solvent dichloromethane, and 61 ± 7% (*v*/*w*) with the polar solvent methanol.

**Figure 1:**
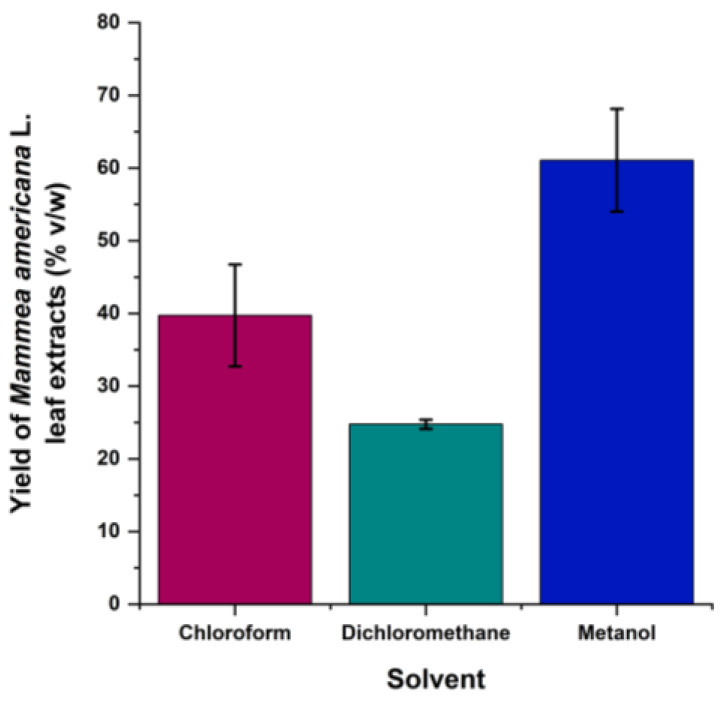
Extraction Yields (%*v*/*w*) with different solvents for *Mammea americana* L. leaf extracts by micro-Soxhlet Extraction. Data in the bar charts is expressed as means ± standard deviation (n = 3).

As seen in Figure 1, methanol showed the highest solvent affinity for extracting the chemical constituents of *Mammea americana* fresh leaves by the extraction method applied. Despite dichloromethane having the lowest affinity for extraction, it had the lowest variability in extraction yield (%), as seen with the error bars representative of the standard deviation.

### 2.2. Phytochemical Profiling by GC-MS

**Figure 2:**
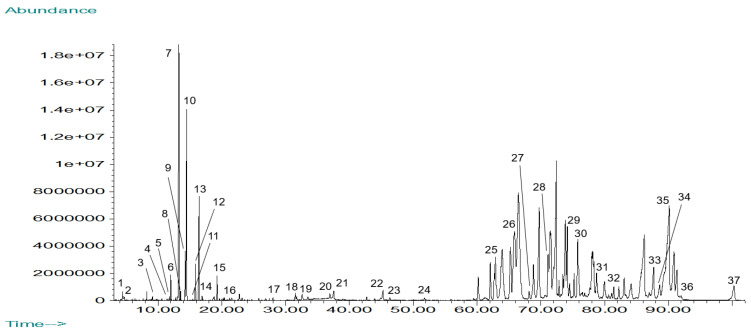
Total Ion Chromatogram for *Mammea americana* chloroform (MAC) leaf extract by GC-MS analysis. Peak identities are listed in Table 1.

The total ion chromatogram for the GC-MS analysis of *Mammea americana* chloroform (MAC) leaf extract is shown in Figure 2. From the separated chromatographic peaks, a total of 37 phytochemical constituents were identified, corresponding to 40.46% of the chromatogram’s total area (Table 1).

The main constituents identified in the MAC extract were mammea-A/AB (9.62%), 6-phenyl-2-[(4,6,8-trimethyl-2-quinazolinyl)amino]-4-pyrimidinol (5.03%), (-)-trans-caryophyllene (4.28%), trans-.beta.-farnesene (3.58%), 10,13-di-t-butyl-14H-benzo[6,7]cyclohepta[1,2-b]naphtho[1,2-d]indole-14-one (2.59%), mammea-B/AC (2.37%), 1,4-diphenyltriphenylene-2,3-diol (1.67%), (E,E)-alpha-farnesene (1.60%), (2,5-diphenyl-1H-pyrrol-3-yl)-(2-naphthalenyl)methanone (1.52%), and mammea-E/BB (1.37%). Identified phytochemical compounds in MAC extract chromatographic profile were classified as sesquiterpenes (24.32%), carboxylic acids (13.51%), coumarins (8.11%), esters (8.11%), alcohols (5.41%), ketones (5.41%), triterpenoids (5.41%), aldehydes (2.70%), amines (2.70%), benzene derivatives (2.70%), diazines (2.70%), diterpenes (2.70%), indoles (2.70%), phenols (2.70%), pyridines (2.70%), pyrroles (2.70%), sesquiterpenoids (2.70%), and steroids (2.70%). Among the phytochemicals identified in the MAC extract, 24 are reported in the literature for having different types of biological activities, representing 64.86% of the extract active compounds. There are compounds with antibacterial (18.92%), anti-inflammatory (16.22%), insecticidal (10.81%), anticancer (8.11%), cytotoxic (8.11%), and antioxidant (5.41%) activities, among other biological activities, including antiviral and analgesic properties. Phytochemicals in MAC extract which are reported in the literature for their antibacterial activity included copaene, (-)-trans-caryophyllene, (+)-δ-cadinene, neophytadiene, hexadecanoic acid, mammea-A/AB, and friedelin [22,23]. Neophytadiene, hexadecanoic acid, mammea-A/AB, and friedelin are also reported to have anti-inflammatory activity, as well as α-humulene and taraxasterol [22,23]. Compounds in MAC extract with insecticidal-related activity included 6-methyl-5-hepten-2-one, (+)-δ-cadinene, the alarm pheromone trans-.beta.-farnesene, and the insect attractant (E,E)-alpha-farnesene. Phytochemicals in MAC extract reported for their anticancer activity included (+)-δ-cadinene, α-humulene, and di-isobutyl phthalate, and for their cytotoxic activity included mammea-E/BB, mammea-A/AB and β-ionone [22,23]. β-ionone and α-tocopherol are reported in the literature for their antioxidant activity [22,23].

**Figure 3:**
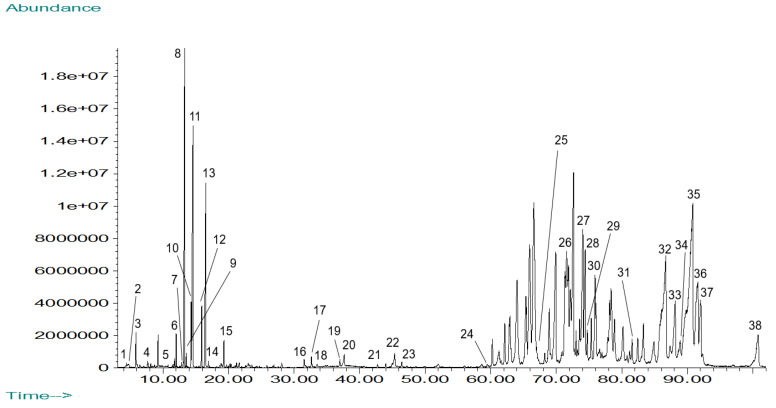
Total Ion Chromatogram for *Mammea americana* dichloromethane (MAD) leaf extract by GC-MS analysis. Peak identities are listed in Table 2.

The total ion chromatogram for the GC-MS analysis of *Mammea americana* dichloromethane (MAD) leaf extract is shown in Figure 3. A total of 38 phytochemical compounds were identified from the separated chromatographic peaks, corresponding to 52.69% of the chromatogram’s total area (Table 2).

The main constituents identified in MAD extract were mammea-A/AB (12.02%), mammea-A/BD (6.96%), mammea-E/BB (4.72%), 2-(1,3-benzodioxol-5-yl)-3-methoxy-6-(pyridin-2-ylmethylsulfanyl)imidazo[1,2-b]pyridazine (3.58%), mammea-B/AB (3.15%), (-)-trans-caryophyllene (3.07%), trans-.beta.-farnesene (2.81%), mammea-B/BC (2.30%), cholestane, ethanone derivative (2.14%), and (E,E)-alpha-farnesene (2.05%). Identified phytochemical compounds in MAD extract chromatographic profile included sesquiterpenes (23.68%), coumarins (13.16%), carboxylic acids (10.53%), hydrocarbons (7.89%), diterpenes (5.26%), esters (5.26%), ketones (5.26%), steroids (5.26%), benzene derivatives (2.63%), diazines (2.63%), indoles (2.63%), phenalenones (2.63%), phenols (2.63%), pyrones (2.63%), quinolones (2.63%), sesquiterpenoids (2.63%), and triterpenoids (2.63%). Among the phytochemicals identified in MAD extract, 26 are reported in the literature for having different types of biological activities, representing 68.42% of the extract active compounds. This includes compounds with antibacterial (23.68%), anti-inflammatory (15.79%), cytotoxic (13.16%), insecticidal (13.16%), anticancer (7.89%), and antioxidant (5.26%) activities, along with other phytochemicals with antifungal and antiviral properties. Phytochemicals in MAD extract reported in the literature for having antibacterial activity included isocaryophyllene, (-)-trans-caryophyllene, (+)-δ-cadinene, neophytadiene, neophytadiene isomer III, hexadecanoic acid, β-sitosterol, mammea-A/AB, and friedelin [22,23]. Compounds reported with anti-inflammatory activity included α-humulene, neophytadiene, neophytadiene isomer III, hexadecanoic acid, mammea-A/AB, and friedelin [22,23]. Mammea-A/AB is also reported in the literature for its cytotoxic activity as well as β-bisabolene, mammea-E/BB, mammea-B/BC, and mammea-A/BD [22,23]. Phytocompounds with insecticidal-related activity included 6-methyl-5-hepten-2-one, (+)-δ-cadinene, the alarm pheromone trans-.beta.-farnesene, the insect attractant (E,E)-alpha-farnesene, and the insect repellent dibutylphthalate [22,23]. (+)-δ-Cadinene is also reported in the literature for having anticancer activity, along with α-humulene and di-isobutyl phthalate. Dihydrodihydroxymaltol and α-tocopherol are reported for their antioxidant activity [22,23].

**Figure 4:**
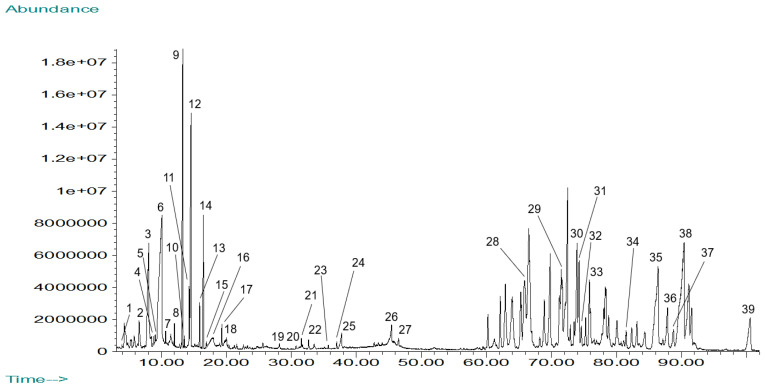
Total Ion Chromatogram for *Mammea americana* methanolic (MAM) leaf extract by GC-MS analysis. Peak identities are listed in Table 3.

The total ion chromatogram for the GC-MS analysis of *Mammea americana* methanolic (MAM) leaf extract is shown in Figure 4. A total of 39 phytochemical constituents were identified from the separated chromatographic peaks, corresponding to 52.81% of the chromatogram’s total area (Table 3).

The main constituents identified in the MAM extract were 5-hydroxymethyl-2-furaldehyde (7.76%), mammea-A/AB (7.52%), mesuol (4.99%), (-)-trans-caryophyllene (3.41%), dihydrodihydroxymaltol (3.39%), trans-.beta.-farnesene (3.28%), lycodoline (3.13%), mammea-B/AB (2.66%), mammea-B/AC (1.83%), and friedelin (1.57%). Identified phytochemical compounds in the MAM extract chromatographic profile included sesquiterpenes (20.51%), carboxylic acids (17.95%), coumarins (12.82%), esters (10.26%), aldehydes (5.13%), alkaloids (5.13%), phenols (5.13%), pyrones (5.13%), diterpenes (2.56%), furans (2.56%), indoles (2.56%), porphyrins (2.56%), sesquiterpenoids (2.56%), steroids (2.56%), and triterpenoids (2.56%). There are 28 compounds among the phytochemicals identified in the MAM extract reported in the literature for having different types of biological activities, representing 71.79% of the extract active compounds. There are phytocompounds with antibacterial (23.08%), anti-inflammatory (15.38%), insecticidal (12.82%), cytotoxic (7.69%), antioxidant (5.13%), and anticancer (2.56%) activities. Other compounds with biological activities included phytocompounds with the potential for regulating glucose levels and with antiviral and antifungal properties. Phytochemicals identified in MAM extract reported in the literature for having antibacterial activity included benzenecarboxylic acid, 5-hydroxymethyl-2-furaldehyde, copaene, (-)-trans-caryophyllene, neophytadiene, hexadecanoic acid, mesuol, mammea-A/AB and friedelin [22,23]. The last five are reported to have anti-inflammatory properties along with α-humulene [22,23]. Phytocompounds identified in MAM leaf extract reported in the literature for their insecticidal-related activity included the acaricide benzenecarboxylic acid, the alarm pheromone trans-.beta.-farnesene, the insect attractant (E,E)-alpha-farnesene, the insect repellent dibutylphthalate, and the coumarin mammea-B/BA [22,23]. Mammea-B/BA, mesuol, and mammea-A/AB are reported to have cytotoxic activity [22,23]. Other phytocompounds identified in MAM extract were dihydrodihydroxymaltol and α-tocopherol, which are reported to have antioxidant activity, and the sesquiterpene with anticancer activity α-humulene [22,23].

**Figure 5:**
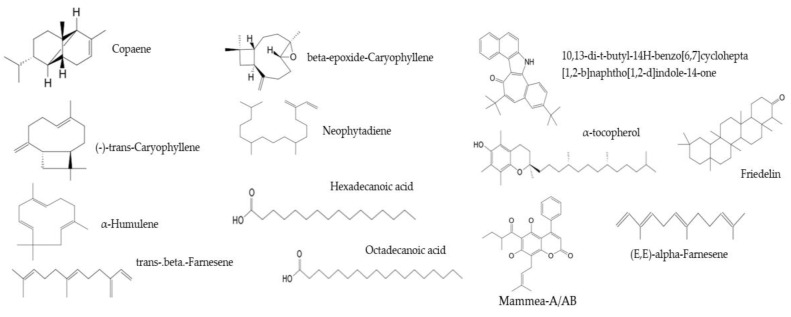
Molecular structures for shared phytochemical constituents in the three analyzed leaf extracts from *Mammea americana* L. (Calophyllaceae) were identified by GC-MS analysis [24].

At least 13 compounds were common to the three different phytochemical profiles of analyzed *Mammea americana* leaf extracts, including mammea-A/AB, (-)-trans-caryophyllene, trans-.beta.-farnesene, 10,13-di-t-butyl-14H-benzo[6,7]cyclohepta[1,2-b]naphtho[1,2-d]indole-14-one, (E,E)-alpha-farnesene, friedelin, α-humulene, α-tocopherol, beta-epoxide-caryophyllene, hexadecanoic acid, copaene, neophytadiene, and octadecanoic acid (Figure 5).

**Figure 6:**
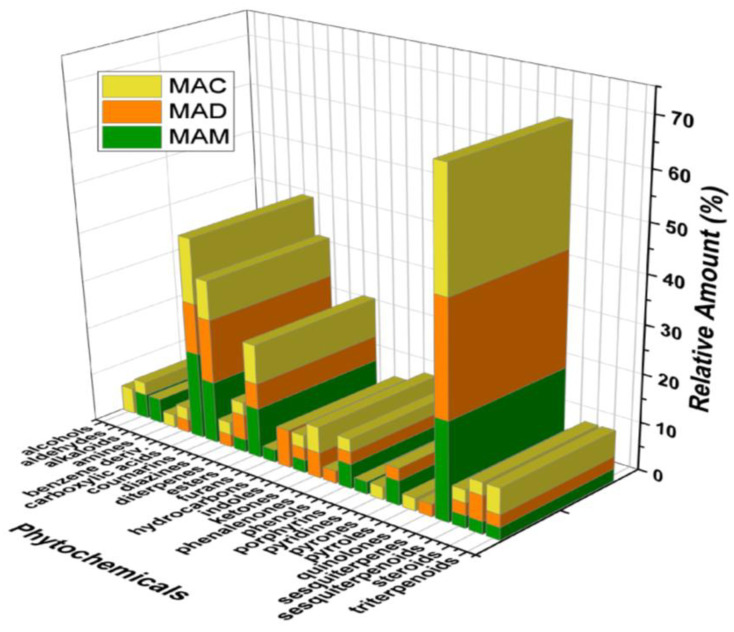
Relative amount (%) for the different classes of phytochemicals present in the three leaf extracts from *Mammea americana* L. (Calophyllaceae).

In general, the three leaf extracts obtained from *Mammea americana* L. (Calophyllaceae) were mainly composed of sesquiterpenes, followed by carboxylic acids, coumarins, esters, diterpenes, phenols, steroids, triterpenoids, indoles, and sesquiterpenoids, in order of decreasing relative amount % (Figure 6).

### 2.3. Brine Shrimp Lethality Bioassays

The bioactivity of the dichloromethane, chloroform, and methanolic extracts of *Mammea americana* L. fresh leaves was evaluated through brine shrimp lethality tests, and the results are summarized in Table 4. The results show that the three leaf extracts tested proved to be bioactive against *Artemia salina* larvae. MAD, MAC, and MAM extracts reached a maximum brine shrimp mortality of 100 ± 0% at 50 μg/mL. Positive control with potassium dichromate (K_2_Cr_2_O_7_) reached 100 ± 0% of brine shrimp mortality at 500 μg/mL, while no mortality of brine shrimps was observed for the negative control with DMSO.

**Figure 7:**
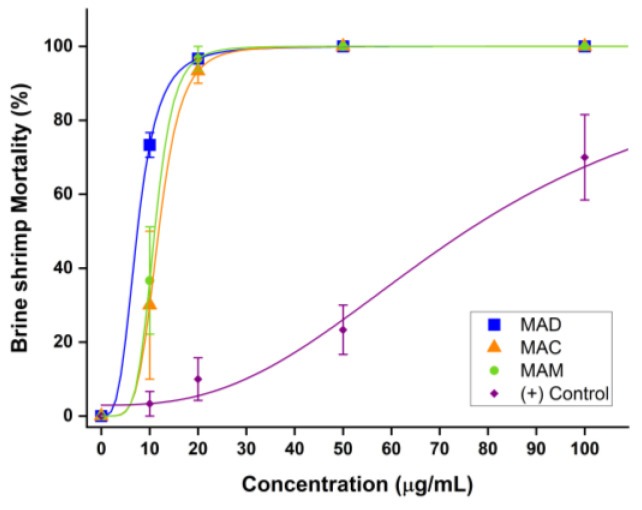
Effect of *Mammea americana* leaf extracts on *Artemia salina* larvae zoomed to 0–100 μg/mL concentration range. Logistic regression was used for curve fitting. Percentages represent the means ± standard error of triplicates. Significant change at *p* ≤ 0.05.

Dose–response curves for the brine shrimp lethality bioassays with the different leaf extracts in Figure 7 are zoomed into the 0–100 μg/mL concentration range for evaluation purposes. These curves show an increase in brine shrimp mortality percentage with increasing extract concentration for MAD, MAC, and MAM extracts, which are much more toxic to brine shrimp than the positive control.

The nonlinear curve fitting with logistic regression used to analyze the curves resulted in a very strong correlation between experimental variables, with coefficients of determination or R^2^ values of 1.00 for the three *Mammea* leaf extracts tested and 0.995 for the positive control dose–response curve (Table 5). The *p*-values obtained were <0.0001, confirming that an effect from the applied treatment was observed for MAD, MAC, and MAM leaf extracts, and for the positive control since Student’s *t*-test resulted in *p* ≤ 0.05, which means that treatment response was significantly different from control.

In terms of the toxicity against *Artemia salina* larvae, all tested *Mammea* leaf extracts were considered highly toxic, with MAD extract having the lowest lethal concentration LC_50_ value of 8.39 (6.55–10.23) μg/mL, followed by MAM with 11.66 (9.90–13.42) μg/mL, and finally MAC with 12.67 (10.84–14.51) μg/mL.

The toxic (+) Control had an LC_50_ of 109.98 (89.19–130.77) μg/mL which is much less toxic compared to the LC_50_ values obtained for MAD, MAM, and MAC leaf extracts. These results confirm the biological activity and the potential cytotoxicity of *Mammea americana* L. leaf extracts due to the high presence of bioactive phytocompounds in their volatile and semi-volatile mixture, which could be further investigated through antibacterial, anti-inflammatory, antioxidant, and/or cancer cell lines-based bioassays. Furthermore, these results correlate with the GC-MS phytochemical profiling, with MAD extract having the highest relative amount of identified cytotoxic compounds (13.16%), compared to MAC (8.11%) and MAM (7.69%) extracts.

### 2.4. Insecticidal Activity Bioassays

The insecticidal activity of the dichloromethane, chloroform, and methanolic extracts of *Mammea americana* L. fresh leaves was evaluated through contact toxicity bioassays, and the results are summarized in Table 6. A quantity of 108 *Ferrisia* insects were subjected to test for each leaf extract treatment, including negative controls with the extraction solvent only. Results demonstrate that the three tested leaf extracts had insecticidal activity against *Ferrisia* sp. (Hemiptera:Pseudococcidae) to a different extent. After 24 h of treatment, MAD extract showed the highest insect mortality with 50 ± 17%, followed by MAM with 44 ± 24%, and then by MAC with 28 ± 6%, at the 10 mg/mL of treatment concentration. Mortality with extracts was lower, compared to the (+) Control, with carbaryl commercial insecticide, which showed an 89 ± 6% insect mortality after 24 h of treatment. After 48 h of treatment, the MAM extract showed the highest insect mortality with 67 ± 10% at 10 mg/mL, followed by MAD and MAC, which had no change in insect mortality from 24 to 48 h at this extract concentration. The (+) Control showed 100 ± 0% insect mortality after 48 h. It is important to note that corrected insect mortality was determined according to Equation (4) only for all treatments prepared in methanol since mortality of insects was observed in the negative controls with this solvent.

**Figure 8:**
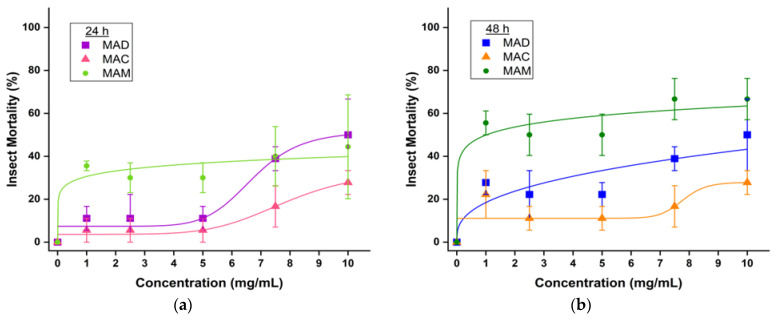
Effect of *Mammea americana* L. leaf extracts on *Ferrisia* sp. insects after (**a**) 24 and (**b**) 48 h of treatment. Logistic regression was used for curve fitting. Percentages represent the means ± standard error of triplicates. Significant change at *p* ≤ 0.05.

Dose–response curves for the insecticidal bioassays, in Figure 8, show that in general, at the lowest concentrations, from 1.0 to 5.0 mg/mL of leaf extract treatment, the mortality rate of insects had almost constant behavior. Then, an increase was observed, from 7.5 to 10.0 mg/mL, with increasing extract concentration.

The nonlinear curve fitting with logistic regression used to analyze the 24 h dose–response curves resulted in a very high correlation between experimental variables, with R^2^ values of 0.911 for MAM, 0.957 for MAD, and 0.961 for MAC (Table 7). The *p*-values obtained were 0.03 for MAM and 0.04 for both MAD and MAC, confirming that an effect from the applied treatment was observed for all tested extracts at 24 h, since Student’s *t*-test for *p* ≤ 0.05 means that the treatment response was significantly different from control. In contrast, the 48 h dose–response curves resulted in a low correlation between experimental variables for MAC, with an R^2^ value of 0.478, in a high correlation for MAD, with an R^2^ of 0.813, and a very high correlation for MAM, with an R^2^ of 0.943. The *p*-values obtained for the 48 h dose–response curves were 0.26 for MAC, 0.09 for MAD, and 0.02 for MAM, which according to Student’s *t*-test, means that the treatment response was significantly different from control only for MAM extract insecticidal bioassay after 48 h.

The obtained data for MAD and MAC at 48 h can be considered not statistically significant since the *p*-values were *p* > 0.05. They should be further investigated to determine if the insect behavior and response toward the applied treatment at lower concentrations caused a departure from correlation in the 48 h dose–response curves.

In terms of the insecticidal effect of the tested *Mammea* leaf extracts against *Ferrisia* sp. insects, the maximum efficacy was obtained for MAM extract with an LC_50_ of 10.80 (5.30–16.31) mg/mL at 24 h that decreased to 5.90 (3.55–8.25) mg/mL at 48 h. Both MAD and MAC extracts also can be considered effective in killing *Ferrisia* sp. insects, with MAD having an LC_50_ of 9.86 (7.31–12.40) mg/mL, followed by MAC, with an LC_50_ of 14.26 (8.14–20.37) mg/mL, after 24 h of applied treatment. These results confirm the potential of *Mammea* leaf extracts to be applied as natural bioinsecticide alternatives for *Ferrisia* insect pest control. Furthermore, the results correlate with the GC-MS phytochemical profiling, with MAD having the highest relative amount of identified insecticidal-related phytocompounds (13.16%), followed closely by MAM (12.82%) and then by MAC (10.81%). The variation in the insecticidal effect of MAM over MAD could be related to a synergistic effect due to the presence of the acaricide benzenecarboxylic acid and the insecticidal coumarin mammea-B/BA in MAM extract.

## 3. Materials and Methods

### 3.1. Plant Material

**Figure 9:**
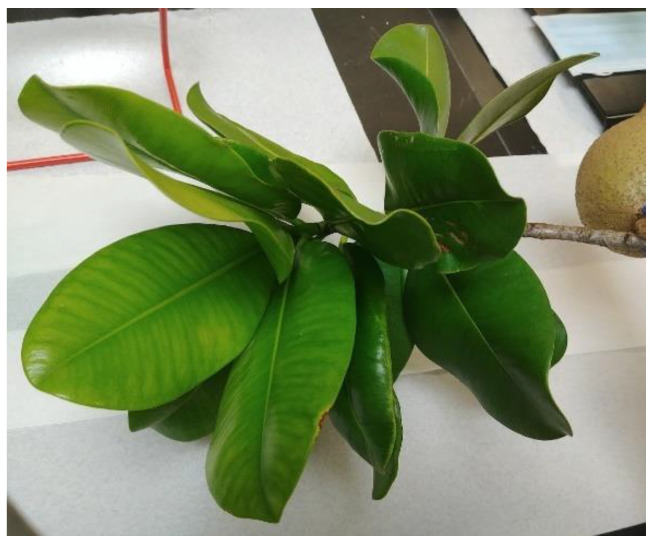
*Mammea americana* L. (Calophyllaceae) fresh leaves collected at Mayagüez, Puerto Rico.

Fresh leaves of *Mammea americana* L. (Calophyllaceae) were collected in October 2018 from a tree located in a mountainous wild area at an altitude of about 372 m above sea level (Latitude 18°21′ N, Longitude −67°04′ W) in Mayagüez, Puerto Rico (Figure 9). The samples were identified and authenticated by Dr. Jeanine Vélez-Gavilán, botanist and taxonomist from the Department of Biology. An equivalent voucher specimen (Accession No. 9064, Barcodes MAPR06600 and MAPR07968, #922, Collector—Atha D. and Sanony T.) was deposited at the MAPR Herbarium, University of Puerto Rico, Mayagüez Campus. The leaves samples were cleaned with distilled water to remove any associated debris and stored at a temperature of −17 °C on freezer plastic bags in a conventional freezer.

### 3.2. Freeze Drying Process

Frozen *Mammea americana* L. (Calophyllaceae) leaves samples were dried using a VirTis Benchtop Freeze Dryer (The Virtis Company, Inc., Gardiner, NY, USA) at a refrigeration temperature of −54.6 °C and vacuum pressure of 140 millitorr for 24 to 28 h. The associated moisture content removed from plant material was 40.6%. After drying, samples were hand-crushed and well-ground using an electric coffee grinder (Mr. Coffee^®^, Newell Brands, Cleveland, OH, USA). Ground plant material was transferred to vials with Teflon-lined caps and stored inside a desiccator at −17 °C in the freezer for the subsequent extraction process.

### 3.3. Preparation of Mammea americana L. Fresh Leaves Extracts

Soxhlet extraction procedure was followed to obtain *Mammea americana* L. fresh leaves extracts. An amount of 0.5 g of dried and ground *Mammea* leaves was transferred to a Whatman 10 × 50 mm cellulose extraction thimble and placed inside the extraction chamber of a micro-Soxhlet extraction apparatus (Kontes Glass Company, Vineland, NJ, USA). An amount of 15 mL of extraction solvent was transferred to a Kontes Glass Company collecting round bottom flask and connected to the micro-Soxhlet apparatus. The solvents for extraction were selected from non-polar to polar to optimize the extraction and identification of the phytochemical constituents of *Mammea americana* leaves. Chloroform (non-polar solvent), dichloromethane (medium-polar solvent), and methanol (polar solvent) HPLC grade from Fisher Scientific Inc. (Ontario, Canada) were used as extraction solvents. The extraction apparatus was connected to an Allihn condenser unit (Kontes Glass Company, Vineland, NJ, USA) continuously supplied with cold water from a circulating cooling bath (Fisher Scientific Isotemp 3016H, Fisher Scientific Inc., Pittsburgh, PA, USA). Extraction was performed for 4 h of constant solvent reflux and siphoning cycles with temperature controlled by a heating mantle (Electrothermal UNIMANTLE 115V, Cadmus Products, Essex, England) connected to a rheostat (POWERSTAT^®^, Superior Electric, Plainville, CT, USA). The process was performed in triplicates with each extraction solvent. After the extraction process, the solvent was completely removed via rotary evaporation using a Buchi RE111 rotary evaporator (BUCHI Corporation, New Castle, DE, USA), and the obtained plant extract was properly weighed to determine extraction yield (%). Extraction yield (%) was calculated by the following formula:

Extraction Yield (%) = (mass of obtained leaves extract/mass of leaves ground sample) × 100(1)

The leaf extract was quantitatively transferred to a 5 mL inner conical vial using the extraction solvent. Following the evaporation of the solvent by refluxing with pure nitrogen to a volume of 1 mL. The inner conical vial with a Teflon-lined cap was sealed with parafilm, covered with aluminum foil, and stored at −4 °C in a refrigerator until further use.

### 3.4. Gas Chromatography-Mass Spectrometry (GC-MS) Analysis

The volatile and semi-volatile constituents of *Mammea americana* L. fresh leaves extracts were analyzed using a Hewlett-Packard Gas Chromatograph, HP6890 (Agilent Technologies Inc., Wilmington, DE, USA), coupled to a Mass Selective Detector, HP5973 (Agilent Technologies Inc., Wilmington, DE, USA). It was equipped with a non-polar SPB-5 poly(5% diphenyl/95% dimethyl siloxane) phase capillary column (30 m × 0.32 mm i.d. × 0.25 μm film thickness; Supelco, Inc., Bellefonte, PA, USA). The injection port was equipped with a 1.5 mm i.d. direct sleeve liner and 11 mm septum and set in splitless mode at a temperature of 225 °C. The oven temperature was initially held at 70 °C for 4 min, followed by an increase at a ramp rate of 10 °C/min until 125 °C, where it was held for 5 min, then followed by an increase at a ramp rate of 2 °C/min to a final temperature of 250 °C, where finally it was held for 25 min. MS detector interface was at a temperature of 180 °C, and the ionization mode was by electron impact with 70 eV, over the range of 35–500 *m*/*z*. Electron multiplier voltage was 1694 V, and the ion source and quadrupole temperature were set at 230 °C and 106 °C, respectively. Ultra-high purity Helium (99.999%) was used as carrier gas at a constant flow rate of 2.2 mL/min with 6.5 psi of initial nominal pressure. *Mammea* leaf extract samples of 1.0 μL were manually injected for their corresponding analysis and chromatographic separation.

### 3.5. Identification of Phytochemical Constituents

The phytochemical constituents of *Mammea americana* fresh leaves extracts were identified based on their retention time (RT) in the total ion chromatograms (TIC) and the fragmentation patterns in the mass spectra of each individual component by comparing with the reference mass spectra in the Wiley 7th and Wiley 10th mass spectral libraries. Match quality values of 90% or above resulted in an excellent match, 80–90% a good, 70–80% a fair, and <60% a poor, as established by the National Institute of Standards and Technology (NIST) general guidelines [26]. For the calculation of linear retention indices (LRI) values, a standard solution of a series of homologous *n*-alkanes C_7_-C_16_ was analyzed under the above GC-MS conditions. LRI for compounds that were not calculated were obtained from the literature, when available, for similar capillary column and temperature ramp conditions. Mass spectra, along with the LRI and compound boiling points, were used as confirmatory criteria for identification.

### 3.6. Brine Shrimp Lethality Bioassays of Mammea americana L. Leaf Extracts

Brine shrimp lethality bioassays were conducted to assess the toxicity of chloroform, dichloromethane, and methanolic extracts from *Mammea americana* L. fresh leaves. The bioassays were performed following the procedure of Meyer et al. 1982 but with modifications [19]. Brine shrimp (*Artemia salina*) eggs (San Francisco Bay Brand, Inc. 8239 Enterprise Dr. Newark, CA 94560, USA) were hatched in a custom-made plastic hatchery filled with seawater, illuminated with a 40 W incandescent light and under constant aeration for 48 h. Sea water for the bioassays was collected near the south coast (Lat. 17°95′ N, Long. −66°84′ W) at Guánica Dry Forest Reserve, Carr. 333, Guayanilla, Puerto Rico. Collected sea water had a pH of 8.60, a conductivity of 49.4 mS/cm, 34.9 g/L of total dissolved solids, a salinity of 36.3 ppt, and a temperature of 25.8 °C. Stock solutions of 20 mg/mL for *Mammea* leaves chloroform extract (MAC), 10 mg/mL for *Mammea* leaves dichloromethane extract (MAD), and 30 mg/mL for *Mammea* leaves methanol extract (MAM) were obtained by diluting 200 mg, 100 mg, and 300 mg of crude extract in 10 mL of DMSO 50%, respectively. Eight concentrations of leaf extract treatment were tested, including 10, 20, 50, 100, 250, 500, 750, and 1000 μg/mL. For the preparation of the bioassay, 4 mL of seawater was added to 10 mL glass vials, and 10 active nauplii shrimps were collected with a Pasteur pipette from the brighter portion of the hatchery and transferred to each vial, different testing concentrations were obtained by adding varying volumes of leaves extract stock with an Eppendorf 4810 autoclavable micropipette to each vial, and finally they were filled to a total volume of 10 mL with seawater. Potassium dichromate (K_2_Cr_2_O_7_ Fisher Scientific Certified ACS ≥99%) was used as a positive control for the bioassays, matching the series of tested concentrations of leaf extracts. Dimethylsulfoxide—DMSO (Fisher Scientific HPLC grade) was used as a negative control at concentrations of 4%, 0.3%, and 0.1% *v*/*v*. All concentrations of leaf extracts and the positive and negative controls were tested in triplicate. After 24 h, the vials were examined against a lighted background by counting the number of brine shrimp that survived in each vial. Then, brine shrimp mortality % was calculated by the equation:

% Mortality = (amount of death brine shrimp in vial/total amount of brine shrimp in vial) × 100.(2)

### 3.7. Insecticidal Activity of Mammea americana L. Leaf Extracts

#### 3.7.1. *Ferrisia* sp. Insects Collection

*Ferrisia* sp. (Hemiptera:Pseudococcidae) insect colonies were collected at the crop field from mango fruit samples infested with *F. virgata* and *F. dasylirii*, at an altitude of 26 m above sea level (Lat. 18°02′ N, Long. −66°52′ W) in the Agricultural Experiment Station—Juana Díaz, Puerto Rico. Infested fruit samples were carefully placed in plastic boxes to avoid squashing *Ferrisia* insects around the fruits and transported to the Laboratory of Entomology. Insects were maintained at a room temperature of 27 ± 2 °C, 65 ± 5 % relative humidity, and an approximate photoperiod of 12:12 h of light:dark cycle.

#### 3.7.2. Contact Toxicity Insecticidal Bioassays

Contact toxicity bioassays were conducted to determine the insecticidal activity of *Mammea americana* L. fresh leaves extracts against adult females and third instar nymphs of *Ferrisia* sp. (Hemiptera:Pseudococcidae). Stock solutions of 20 mg/mL, 10 mg/mL, and 30 mg/mL were obtained for the chloroform (MAC), dichloromethane (MAD), and methanol (MAM) leaf extracts, respectively, due to extraction yields. These stocks were prepared using the extraction solvent as dilution media. Five diluted solutions were prepared from each extract stock solution with concentrations of 1.0, 2.5, 5.0, 7.5, and 10.0 mg/mL. Following the methodology of Zhao et al. (2012) with modifications, Whatman No. 4 filter paper discs of 5.5 cm in diameter were placed in 5 × 0.9 cm Petri dishes [27,28,29]. A potato tuber piece of an average size of 3 × 1.5 cm and weight of 0.5 to 1.5 g was used as a food source for the insects by placing it over the filter paper. Following the methodology of Pinto et al. (2015) with modifications, 1 mL of diluted leaf extract solution was applied by distributing it through the potato tuber piece, filter paper, and the Petri dish lid [30,31]. The solvent was allowed to evaporate under the Thermo Scientific laboratory bench fume hood (Thermo Fisher Scientific Inc., Waltham, MA, USA), running at low speed for 5 min to dichloromethane, 10 min to chloroform, and 15 min to methanol [30,31]. Then, six insects were exposed to the applied treatment by placing three insects over the potato tuber piece and three over the filter paper area. Negative control dishes were prepared the same way, but 1 mL of the extraction solvent was applied as treatment. Positive control dishes were also prepared using 1 mL of the commercial insecticide Sevin Gardentech^®^ Ready-To-Use Bug Killer (Tech Pac, LLC, Covington, GA, USA) as treatment, whose main ingredient is carbaryl (0.126%). Each leaves extract treatment dilution and the negative controls were tested in triplicates. The bioassays were conducted at a room temperature of 27 ± 2 °C and relative humidity of 65 ± 5%. Insect mortality was recorded after 24 and 48 h of exposure to treatment [32]. Death insects were counted under the magnification of a Nikon SMZ800 stereomicroscope (Nikon Instruments Inc., Melville, NY, USA) based on the mobility of body parts and changes in the color of the body. Insect mortality % was calculated by the equation [32]:

% Mortality = (amount of dead insect in Petri dish/total amount of insects in Petri dish) × 100(3)

when the mortality of insects was observed in the negative controls, corrected insect mortality was calculated by the following formula described by Schneider–Orelli and Puntener and applied by Roddee et al. 2020 [33]:

Corrected Mortality % = [(Mortality % in treatment − Mortality % in control)/(100 − Mortality % in control)] × 100(4)

### 3.8. Statistical Analysis

Experiments were carried out in triplicate, and the values are expressed as the mean ± standard deviation (SD) and/or mean ± standard error (SE). All bioassay dose–response curves of mortality percentage against leaves extract treatment concentration was analyzed by a nonlinear curve fitting following a logistic regression with the Levenberg–Marquardt (L-M) algorithm, which is the standard nonlinear least square iterative procedure that combines the Gauss–Newton method and the steepest descent method, to determine the coefficient of determination. A one-way ANOVA test was used to analyze brine shrimp larvae and insect mortality, followed by a Student’s *t*-test for *p*-values. The null hypothesis is rejected when *p* ≤ 0.05, meaning that results are significantly different from the control, while *p* > 0.05 means that results are similar to the control or the null hypothesis is true. Probit analysis, which transforms the sigmoidal dose–response curves to a straight line through maximum likelihood, was used to calculate LC_50_ values with 95% confidence limits (CL) for both the brine shrimp lethality bioassays and for the insecticidal activity bioassays. OriginLab Pro 2024b software was used to conduct all statistical analyses.

## 4. Conclusions

Results obtained from this study demonstrate that the three leaf extracts from the folkloric medicinal plant, *Mammea americana* L. (Calophyllaceae), are a source of biologically active phytochemicals with a great potential for different applications because of their potential cytotoxicity and insecticidal activities. Brine shrimp lethality bioassays proved that the three tested extracts are highly bioactive or toxic, providing a basis for further research into the antibacterial, anti-inflammatory, and anticancer properties of the bioactive components of *Mammea americana* leaves. Insecticidal activity bioassays confirmed that the three extracts are potential candidates for use as green botanical insecticides or environmentally friendly alternative bioinsecticides against *Ferrisia* sp. (Hemiptera:Pseudococcidae) colonies. It is important to note that the results obtained with the brine shrimp lethality bioassays and the insecticidal activity bioassays correlate well with the GC-MS analysis and phytochemical profiling, being MAD and MAM, the extracts with the highest amount of identified cytotoxic and insecticidal compounds, the most toxic against *Artemia salina*, and having the highest insecticidal effect against *Ferrisia* sp. insects. However, the toxicity and/or insecticidal effectiveness of tested extracts cannot be attributed to one or a few bioactive phytocompounds since both major and minor phytochemicals in the extract mixture can synergistically contribute to any biological activity [34]. Further testing of the potential of these leaf extracts for their use against other insects of agricultural importance or through fumigant or repellent activity bioassays should be conducted to be implemented as an alternative over synthetic or harmful toxic chemicals that currently are used to kill or repel pests from crops.

## Figures and Tables

**Table 1:** GC-MS phytochemical profile for *Mammea americana* chloroform (MAC) leaf extract.

ID No.	Phytocompound	RT (min)	Area (%)	M.F.	M.W.	LRI [21]	Classification
1	6-Methyl-5-hepten-2-one	4.47	0.13	C_8_H_14_O	126	711	ketone
2	4-Hepten-1-ol	4.70	0.08	C_7_H_14_O	114	713	alcohol
3	3-Hydroxy-1-cyclohexene-1-carbaldehyde	9.16	0.05	C_7_H_10_O_2_	126	1300	aldehyde
4	1,2-dihydro-1,5,8-trimethylnaphthalene	11.44	0.02	C_13_H_16_	172	1366	benzene derivative
5	(+)-Cyclosativene	11.79	0.05	C_15_H_24_	204	1376	sesquiterpene
6	Copaene	12.01	0.22	C_15_H_24_	204	1382	sesquiterpene
7	(-)-trans-Caryophyllene	13.27	4.28	C_15_H_24_	204	1405	sesquiterpene
8	Bergamotene	13.54	0.10	C_15_H_24_	204	1426	sesquiterpene
9	α-Humulene	14.27	0.58	C_15_H_24_	204	1447	sesquiterpene
10	trans-.beta.-Farnesene	14.51	3.58	C_15_H_24_	204	1454	sesquiterpene
11	β-Ionone	15.45	0.02	C_13_H_20_O	192	1481	ketone
12	(3Z,6E)-alpha.-Farnesene	15.86	0.45	C_15_H_24_	204	1493	sesquiterpene
13	(E,E)-alpha-Farnesene	16.43	1.60	C_15_H_24_	204	1507	sesquiterpene
14	(+)-δ-Cadinene	16.92	0.05	C_15_H_24_	204	1519	sesquiterpene
15	beta-epoxide-Caryophyllene	19.26	0.35	C_15_H_24_O	220	1573	sesquiterpenoid
16	Diethyl Phthalate	19.92	0.02	C_12_H_14_O_4_	222	1589	ester
17	Myristic acid	28.03	0.06	C_14_H_28_O_2_	228	1765 ^lit^	carboxylic acid
18	Neophytadiene	31.52	0.14	C_20_H_38_	278	1838 ^lit^	diterpene
19	di-Isobutyl Phthalate	32.60	0.14	C_16_H_22_O_4_	278	1877 ^lit^	ester
20	Butyl Isobutyl Phthalate	36.94	0.09	C_16_H_22_O_4_	278	1924 ^lit^	ester
21	Hexadecanoic acid	37.55	0.24	C_16_H_32_O_2_	256	1973 ^lit^	carboxylic acid
22	Linoleic acid	44.91	0.07	C_18_H_32_O_2_	280	2104 ^lit^	carboxylic acid
23	Octadecanoic acid	46.33	0.05	C_18_H_36_O_2_	284	2172 ^lit^	carboxylic acid
24	2-(3-Methylbenzyl)-1-naphthoic acid	51.76	0.12	C_19_H_16_O_2_	276	N/A	carboxylic acid
25	(2,5-diphenyl-1H-pyrrol-3-yl)-(2-naphthalenyl)methanone	62.87	1.52	C_27_H_19_NO	373	N/A	pyrrole
26	6-phenyl-2-[(4,6,8-trimethyl-2-quinazolinyl)amino]-4-pyrimidinol	65.84	5.03	C_21_H_19_N_5_O	357	N/A	diazine
27	3,3′:5,3″-bis(dimethylene)-2,6-di(1′,8′-naphthyrid-2′-yl)pyridine	68.17	0.26	C_25_H_17_N_5_	387	N/A	pyridine
28	Mammea-E/BB	71.12	1.37	C_24_H_30_O_7_	430	N/A	coumarin
29	Mammea-B/AC	74.16	2.37	C_21_H_26_O_5_	358	N/A	coumarin
30	10,13-Di-t-butyl-14H-benzo[6,7]cyclohepta[1,2-b]naphtho[1,2-d]indole-14-one	75.78	2.59	C_29_H_29_NO	407	N/A	indole
31	N,N′-bis[ethoxy(phenyl)phosphoryl]ethane-1,2-diamine	78.73	1.12	C_18_H_26_N_2_O_4_P_2_	396	N/A	amine
32	α-Tocopherol	81.44	0.40	C_29_H_50_O_2_	430	3130 ^lit^	phenol
33	1,4-Diphenyltriphenylene-2,3-diol	87.68	1.67	C_30_H_20_O_2_	412	N/A	alcohol
34	γ-Sitosterol	88.62	0.86	C_29_H_50_O	414	N/A	steroid
35	Mammea-A/AB	90.12	9.62	C_25_H_26_O_5_	406	N/A	coumarin
36	Taraxasterol	91.99	0.19	C_30_H_50_O	426	N/A	triterpenoid
37	Friedelin	100.25	0.97	C_30_H_50_O	426	3510 ^lit^	triterpenoid
Identified Total Area %			40.46				

RT = retention time, M.F. = molecular formula, M.W. = molecular weight, LRI = linear retention index, ^lit^ = LRI obtained from literature, N/A = not available.

**Table 2:** GC-MS phytochemical profile for *Mammea americana* dichloromethane (MAD) leaf extract.

ID No.	Phytocompound	RT (min)	Area (%)	M.F.	M.W.	LRI [21]	Classification
1	6-Methyl-5-hepten-2-one	4.45	0.07	C_8_H_14_O	126	710	ketone
2	trans-3-Hexenoic acid	4.80	0.07	C_6_H_10_O_2_	114	714	carboxylic acid
3	2-Chlorocyclohexanone	5.86	0.29	C_6_H_9_ClO	132	726	ketone
4	Dihydrodihydroxymaltol	7.68	0.07	C_6_H_8_O_4_	144	1130 ^lit^	pyrone
5	Naphthalene,1,2-dihydro-1,5,8-trimethyl-	11.45	0.02	C_13_H_16_	172	1366	benzene derivative
6	Copaene	12.02	0.17	C_15_H_24_	204	1382	sesquiterpene
7	Isocaryophyllene	12.83	0.04	C_15_H_24_	204	1405	sesquiterpene
8	(-)-trans-Caryophyllene	13.30	3.07	C_15_H_24_	204	1419	sesquiterpene
9	β-Bisabolene	13.56	0.08	C_15_H_24_	204	1426	sesquiterpene
10	α-Humulene	14.29	0.42	C_15_H_24_	204	1447	sesquiterpene
11	trans-.beta.-Farnesene	14.56	2.81	C_15_H_24_	204	1455	sesquiterpene
12	(3Z,6E)-alpha.-Farnesene	15.90	0.44	C_15_H_24_	204	1494	sesquiterpene
13	(E,E)-alpha-Farnesene	16.52	2.05	C_15_H_24_	204	1509	sesquiterpene
14	(+)-δ-Cadinene	16.96	0.04	C_15_H_24_	204	1520	sesquiterpene
15	beta-epoxide-Caryophyllene	19.30	0.24	C_15_H_24_O	220	1574	sesquiterpenoid
16	Neophytadiene	31.55	0.09	C_20_H_38_	278	1838 ^lit^	diterpene
17	di-Isobutyl Phthalate	32.65	0.12	C_16_H_22_O_4_	278	1877 ^lit^	ester
18	Neophytadiene, Isomer III	33.52	0.04	C_20_H_38_	278	1883 ^lit^	diterpene
19	Dibutylphthalate	36.99	0.07	C_16_H_22_O_4_	278	1907 ^lit^	ester
20	Hexadecanoic acid	37.66	0.22	C_16_H_32_O_2_	256	1973 ^lit^	carboxylic acid
21	3-Eicosene	42.73	0.04	C_20_H_40_	280	N/A	hydrocarbon
22	Linoleic acid	45.00	0.07	C_18_H_32_O_2_	280	2104 ^lit^	carboxylic acid
23	Octadecanoic acid	46.44	0.05	C_18_H_36_O_2_	284	2172 ^lit^	carboxylic acid
24	1-Docosene	59.48	0.06	C_22_H_44_	308	2194 ^lit^	hydrocarbon
25	Cyclotetracosane	67.00	0.49	C_24_H_48_	336	2589 ^lit^	hydrocarbon
26	Mammea-E/BB	71.58	4.72	C_24_H_30_O_7_	430	N/A	coumarin
27	Mammea-B/AB	74.05	3.15	C_22_H_28_O_5_	372	N/A	coumarin
28	Mammea-B/BC	74.42	2.30	C_21_H_26_O_5_	358	N/A	coumarin
29	Herqueinone	74.73	0.58	C_20_H_20_O_7_	372	N/A	phenalenone
30	10,13-Di-t-butyl-14H-benzo [6,7]cyclohepta[1,2-b]naphtho[1,2-d]indole-14-one	75.92	1.59	C_29_H_29_NO	407	N/A	indole
31	α-Tocopherol	81.57	0.49	C_29_H_50_O_2_	430	3130 ^lit^	phenol
32	Mammea-A/BD	86.70	6.96	C_24_H_24_O_5_	392	N/A	coumarin
33	Cholestane, ethanone derivative	88.15	2.14	C_29_H_48_O	412	N/A	steroid
34	β-Sitosterol	88.91	0.95	C_29_H_50_O	414	3187 ^lit^	steroid
35	Mammea-A/AB	90.83	12.02	C_25_H_26_O_5_	406	N/A	coumarin
36	2-(1,3-Benzodioxol-5-yl)-3-methoxy-6-(pyridin-2-ylmethylsulfanyl)imidazo[1,2-b]pyridazine	91.60	3.58	C_20_H_16_N_4_O_3_S	392	N/A	diazine
37	1,3-diphenyl-4-[(phenylmethyl)amino]-5,6,7,8-tetrahydroquinolin-2-one	92.04	1.67	C_28_H_26_N_2_O	406	N/A	quinolone
38	Friedelin	100.79	1.41	C_30_H_50_O	426	3510 ^lit^	triterpenoid
	Identified Total Area %		52.69				

RT = retention time, M.F. = molecular formula, M.W. = molecular weight, LRI = linear retention index, ^lit^ = LRI obtained from literature, N/A = not available.

**Table 3:** GC-MS phytochemical profile for *Mammea americana* methanolic (MAM) leaf extract.

ID No.	Phytocompound	RT (min)	Area (%)	M.F.	M.W.	LRI [21]	Classification
1	5-Methylfurfural	3.95	0.06	C_6_H_6_O_2_	110	705	aldehyde
2	2-Furoic acid methyl ester	6.58	0.63	C_6_H_6_O_3_	126	980 ^lit^	ester
3	Dihydrodihydroxymaltol	8.08	3.39	C_6_H_8_O_4_	144	1130 ^lit^	pyrone
4	Benzenecarboxylic acid	8.47	0.27	C_7_H_6_O_2_	122	1170 ^lit^	carboxylic acid
5	4H-Pyran-4-one, 3,5-dihydroxy-2-methyl-	8.76	0.13	C_6_H_6_O_4_	142	1188 ^lit^	pyrone
6	5-Hydroxymethyl-2-furaldehyde	10.07	7.76	C_6_H_6_O_3_	126	1326	furan
7	2-Methoxy-4-vinyl-phenol	10.66	0.20	C_9_H_10_O_2_	150	1343	phenol
8	Copaene	12.02	0.20	C_15_H_24_	204	1382	sesquiterpene
9	(-)-trans-Caryophyllene	13.28	3.41	C_15_H_24_	204	1418	sesquiterpene
10	β-Sesquiphellandrene	13.56	0.13	C_15_H_24_	204	1426	sesquiterpene
11	α-Humulene	14.29	0.64	C_15_H_24_	204	1447	sesquiterpene
12	trans-.beta.-Farnesene	14.54	3.28	C_15_H_24_	204	1455	sesquiterpene
13	trans-α-Bergamotene	15.89	0.46	C_15_H_24_	204	1493	sesquiterpene
14	(E,E)-alpha-Farnesene	16.47	1.47	C_15_H_24_	204	1508	sesquiterpene
15	β-Cadinene	16.94	0.10	C_15_H_24_	204	1519	sesquiterpene
16	3-Hydroxy-benzoic acid	17.95	1.17	C_7_H_6_O_3_	138	1543	carboxylic acid
17	beta-epoxide-Caryophyllene	19.31	0.31	C_15_H_24_O	220	1574	sesquiterpenoid
18	4-Hydroxy-3-methoxybenzoic acid	20.00	0.25	C_8_H_8_O_4_	168	1590	carboxylic acid
19	Myristic acid	28.20	0.11	C_14_H_28_O_2_	228	1765 ^lit^	carboxylic acid
20	9H-Indeno[2,1-c]pyridin-9-one	30.75	0.05	C_12_H_7_NO	181	N/A	alkaloid
21	Neophytadiene	31.54	0.14	C_20_H_38_	278	1838 ^lit^	diterpene
22	Butyl Isobutyl Phthalate	32.65	0.14	C_16_H_22_O_4_	278	1924 ^lit^	ester
23	Methyl hexadecanoate	35.69	0.05	C_17_H_34_O_2_	270	1928 ^lit^	ester
24	Dibutylphthalate	36.99	0.08	C_16_H_22_O_4_	278	1970 ^lit^	ester
25	Hexadecanoic acid	37.75	0.44	C_16_H_32_O_2_	256	1973 ^lit^	carboxylic acid
26	(Z)-9,17-Octadecadienal	45.41	0.91	C_18_H_32_O	264	1997 ^lit^	aldehyde
27	Octadecanoic acid	46.48	0.14	C_18_H_36_O_2_	284	2172 ^lit^	carboxylic acid
28	Lycodoline	65.85	3.13	C_16_H_25_NO_2_	263	N/A	alkaloid
29	3-[(3-nitro-4-pyridinyl)amino]benzoic acid	71.67	1.18	C_12_H_9_N_3_O_4_	259	N/A	carboxylic acid
30	Mammea-B/AB	73.93	2.66	C_22_H_28_O_5_	372	N/A	coumarin
31	Mammea-B/AC	74.24	1.83	C_21_H_26_O_5_	358	N/A	coumarin
32	Mammea-B/BA	74.60	0.46	C_22_H_28_O_5_	372	N/A	coumarin
33	10,13-Di-t-butyl-14H-benzo[6,7]cyclohepta[1,2-b]naphtho[1,2-d]indole-14-one	75.84	1.36	C_29_H_29_NO	407	N/A	indole
34	α-Tocopherol	81.49	0.38	C_29_H_50_O_2_	430	3130 ^lit^	phenol
35	Mesuol	86.39	4.99	C_24_H_24_O_5_	392	N/A	coumarin
36	13,17-Diethyl-12,18-dimethyl-21,22-dioxaoxophlorin	87.85	1.31	C_26_H_24_N_2_O_3_	412	N/A	porphyrin
37	γ-Sitosterol	88.74	0.50	C_29_H_50_O	414	N/A	steroid
38	Mammea-A/AB	90.38	7.52	C_25_H_26_O_5_	406	N/A	coumarin
39	Friedelin	100.56	1.57	C_30_H_50_O	426	3510 ^lit^	triterpenoid
	Identified Total Area %		52.81				

RT = retention time, M.F. = molecular formula, M.W. = molecular weight, LRI = linear retention index, ^lit^ = LRI obtained from literature, N/A = not available.

**Table 4:** Mean mortality percentage for *Artemia salina* larvae exposed to *Mammea americana* leaf extracts and controls.

Mean Brine Shrimp Mortality (%) (M ± SE)				
Conc. (μg/mL)	MAD	MAC	MAM	(+) Control K_2_Cr_2_O_7_
10	73 ± 3	30 ± 20	37 ± 15	3 ± 3
20	97 ± 3	93 ± 3	97 ± 3	10 ± 6
50	100 ± 0	100 ± 0	100 ± 0	23 ± 7
100	100 ± 0	100 ± 0	100 ± 0	70 ± 12
250	100 ± 0	100 ± 0	100 ± 0	90 ± 6
500	100 ± 0	100 ± 0	100 ± 0	100 ± 0
750	100 ± 0	100 ± 0	100 ± 0	100 ± 0
1000	100 ± 0	100 ± 0	100 ± 0	100 ± 0
Conc. (%*v*/*v*)	(-) Control DMSO			
0.1	0			
0.3	0			
4	0			

Percentages are means ± standard error (*n* = 30). DMSO—Dimethylsulfoxide.

**Table 5:** Toxicity of *Mammea americana* L. leaf extracts against *Artemia salina* larvae.

*Mammea* Leaves Extract Treatment	LC_50_ µg/mL (95% CL)	Toxicity Profile *	Regression Equation	R^2^	χ^2^ (d*f* = 5)	*p*-Value
MAD	8.39 (6.55–10.23)	Highly toxic	$y=\frac{7.77\;\times \;10^{-5}-100.03}{{1+\left(x/7.43\right)}^{3.39}}+100.03$	1.00	0.004	<0.0001
MAC	12.67 (10.84–14.51)	Highly toxic	$y=\frac{1.22\;\times \;10^{-4}-\;100.01}{{1+\left(x/11.84\right)}^{5.03}}+100.01$	1.00	0.001	<0.0001
MAM	11.66 (9.90–13.42)	Highly toxic	$y=\frac{6.97\;\times \;10^{-6}-\;100.00}{{1+\left(x/11.02\right)}^{5.65}}+100.00$	1.00	0.0001	<0.0001
(+) Control K_2_Cr_2_O_7_	109.98 (89.19–130.77)	Toxic	$y=\frac{2.96\;-\;99.04}{{1+\left(x/76.71\right)}^{2.69}}+99.04$	0.995	15.02	<0.0001
(-) Control DMSO	0	Non toxic	0	0	0	0

Student’s *t*-test for *p* ≤ 0.05 is significantly different from control. * Score for LC_50_: Highly toxic < 20 μg/mL, Toxic ≤ 1000 μg/mL, Non-toxic > 1000 μg/mL [25]. LC = lethal concentration, CL = confidence limit, R^2^ = coefficient of determination, χ^2^ = reduced chi-square, d*f* = degrees of freedom, DMSO—Dimethylsulfoxide.

**Table 6:** Mean mortality (%) for *Ferrisia* sp. insects exposed to *Mammea americana* L. leaf extracts at 24 and 48 h of treatment.

	Mean Insect Mortality (%) (M ± SE)							
Conc. (mg/mL)	MAD 24 h	MAC 24 h	MAM 24 h	(+) Control 24 h	MAD 48 h	MAC 48 h	MAM 48 h	(+) Control 48 h
1.0	11 ± 6	6 ± 6	36 ± 2	89 ± 6	28 ± 6	22 ± 11	56 ± 6	100 ± 0
2.5	11 ± 11	6 ± 6	30 ± 7		22 ± 11	11 ± 6	50 ± 10	
5.0	11 ± 6	6 ± 6	30 ± 7		22 ± 6	11 ± 6	50 ± 10	
7.5	39 ± 6	17 ± 10	40 ± 14		39 ± 6	17 ± 10	67 ± 10	
10.0	50 ± 17	28 ± 6	44 ± 24		50 ± 17	28 ± 6	67 ± 10	
(-) Control	0	0	0		0	0	0	
1 mL solvent								

Percentages are means ± standard error (*n* = 18). The negative control treatment applied was 1 mL of extraction solvent used. The positive control treatment applied was 1 mL of Sevin Gardentech^®^ Ready-To-Use Bug Killer.

**Table 7:** Insecticidal efficacy of *Mammea americana* L. leaf extracts against *Ferrisia* sp. insects.

*Mammea* Leaves Extract Treatment	Exposure Time	LC_50_ mg/mL (95% CL)	Regression Equation	R^2^	χ^2^ (d*f* = 2)	*p*-Value
MAD	24 h	9.86 (7.31–12.40)	$y=\frac{7.39-51.71}{{1+\left(x/6.71\right)}^{8.07}}+51.71$	0.957	41.10	0.04
	48 h	10.00 (6.14–13.85)	$y=\frac{1.41-344,718.32}{{1+\left(x/1.05\;\times 10^{11}\right)}^{\;0.39}}+344,718.32$	0.813	135.35	0.09
MAC	24 h	14.26 (8.14–20.37)	$y=\frac{3.66-33.72}{{1+\left(x/7.86\right)\;}^{5.83}}+33.72$	0.961	10.23	0.04
	48 h	19.87 (1.82–37.93)	$y=\frac{11.11-27.91}{{1+\left(x/7.78\right)\;}^{19.37}}+27.91$	0.478	123.46	0.26
MAM	24 h	10.80 (5.30–16.31)	$y=\frac{0.08-1921.61}{{1+\left(x/1.99\;\times 10^{15}\right)}^{\;0.12}}+1921.61$	0.911	55.04	0.03
	48 h	5.90 (3.55–8.25)	$y=\frac{0.08-1875.94}{{1+\left(x/3.22\;\times 10^{14}\right)}^{\;0.11}}+1875.94$	0.943	87.00	0.02

Student’s *t*-test for *p* ≤ 0.05 is significantly different from control. LC = lethal concentration, CL = confidence limit, R^2^ = coefficient of determination, χ^2^ = reduced chi-square, d*f* = degrees of freedom.

## Data Availability

Data are contained within the article and Supplementary Materials.

## Supplementary Materials

The following supporting information can be downloaded at: https://www.mdpi.com/article/10.3390/plants14010021/s1, Table S1: GC-MS phytochemical profile for *Mammea americana* chloroform (MAC) leaf extract (full version); Table S2: GC-MS phytochemical profile for *Mammea americana* dichloromethane (MAD) leaf extract (full version); Table S3: GC-MS phytochemical profile for *Mammea americana* methanolic (MAM) leaf extract (full version).
