# Supplementary material for: Phytochemical Profiling, Bioactivity, and Insecticidal Effectiveness of Mammea americana L. Leaf Extracts Against Ferrisia sp
Source: Plants (Basel). 2024 Dec 25;14(1):21. doi: 10.3390/plants14010021 (PMC11723013; doi:10.3390/plants14010021)
Supplement: Supplementary file 1 [file plants-14-00021-s001.zip › plants-3376032-supplementary.pdf]

## Supplementary Information

# Phytochemical Profiling, Bioactivity, and Insecticidal Effectiveness of *Mammea americana* L. Leaf Extracts Against *Ferrisia* sp.

Mike Vázquez-Torres [1,*], Nilka Rivera-Portalatín [1] and Irma Cabrera-Asencio [2]

**Table S1.** GC-MS phytochemical profile for *Mammea americana* chloroform (MAC) leaf extract (full version).

| ID No. | Phytocompound | RT (min) | Area (%) | M.F. | M.W. | LRI [21] | CAS No. | Match Quality (%) | Classification | Biological Activity [22,23] |
|---|---|---|---|---|---|---|---|---|---|---|
| 1 | 6-Methyl-5-hepten-2-one | 4.47 | 0.13 | $C_8H_{14}O$ | 126 | 711 | 110-93-0 | 87 | ketone | insecticidal, alarm pheromone plant metabolite |
| 2 | 4-Hepten-1-ol | 4.70 | 0.08 | $C_7H_{14}O$ | 114 | 713 | 20851-55-2 | 76 | alcohol | - |
| 3 | 3-Hydroxy-1-cyclohexene-1-carbaldehyde | 9.16 | 0.05 | $C_7H_{10}O_2$ | 126 | 1300 | 67252-14-6 | 80 | aldehyde | - |
| 4 | 1,2-dihydro-1,5,8-trimethylnaphthalene | 11.44 | 0.02 | $C_{13}H_{16}$ | 172 | 1366 | 4506-36-9 | 80 | benzene derivative | - |
| 5 | (+)-Cyclosativene | 11.79 | 0.05 | $C_{15}H_{24}$ | 204 | 1376 | 22469-52-9 | 83 | sesquiterpene | - |
| 6 | Copaene | 12.01 | 0.22 | $C_{15}H_{24}$ | 204 | 1382 | 3856-25-5 | 99 | sesquiterpene | antibacterial |
| 7 | (-)-trans-Caryophyllene | 13.27 | 4.28 | $C_{15}H_{24}$ | 204 | 1405 | 87-44-5 | 99 | sesquiterpene | sedative, antibacterial flavoring agent |
| 8 | Bergamotene | 13.54 | 0.10 | $C_{15}H_{24}$ | 204 | 1426 | 15438-93-4 | 93 | sesquiterpene | plant metabolite |
| 9 | α-Humulene | 14.27 | 0.58 | $C_{15}H_{24}$ | 204 | 1447 | 6753-98-6 | 97 | sesquiterpene | anticarcinogenic, anti-inflammatory flavoring agent |
| 10 | trans-.beta.-Farnesene | 14.51 | 3.58 | $C_{15}H_{24}$ | 204 | 1454 | 502-60-3 | 97 | sesquiterpene | alarm pheromone |
| 11 | β-Ionone | 15.45 | 0.02 | $C_{13}H_{20}O$ | 192 | 1481 | 14901-07-6 | 70 | ketone | induces cell growth inhibition apoptosis in human colon cancer cells, antioxidant, flavoring agent |
| 12 | (3Z,6E)-alpha.-Farnesene | 15.86 | 0.45 | $C_{15}H_{24}$ | 204 | 1493 | 26560-14-5 | 91 | sesquiterpene | - |
| 13 | (E,E)-alpha-Farnesene | 16.43 | 1.60 | $C_{15}H_{24}$ | 204 | 1507 | 502-61-4 | 95 | sesquiterpene | codling moth attractant flavoring agent |
| 14 | (+)-δ-Cadinene | 16.92 | 0.05 | $C_{15}H_{24}$ | 204 | 1519 | 483-76-1 | 92 | sesquiterpene | antibacterial, insecticidal, anticancer |
| 15 | beta-epoxide-Caryophyllene | 19.26 | 0.35 | $C_{15}H_{24}O$ | 220 | 1573 | 1139-30-6 | 94 | sesquiterpenoid | flavoring agent |
| 16 | Diethyl Phthalate | 19.92 | 0.02 | $C_{12}H_{14}O_4$ | 222 | 1589 | 84-66-2 | 93 | ester | chemotactic factor |
| 17 | Myristic acid | 28.03 | 0.06 | $C_{14}H_{28}O_2$ | 228 | 1765 [lit] | 544-63-8 | 70 | carboxylic acid | defoaming agent |
| 18 | Neophytadiene | 31.52 | 0.14 | $C_{20}H_{38}$ | 278 | 1838 [lit] | 504-96-1 | 95 | diterpene | anti-inflammatory, antimicrobial |

| | | | | | | | | | | |
|---|---|---|---|---|---|---|---|---|---|---|
| 19 | di-Isobutyl Phthalate | 32.60 | 0.14 | $C_{16}H_{22}O_4$ | 278 | 1877 $^{lit}$ | 84-69-5 | 83 | ester | plant and algal metabolite teratogenic agent, PPAR modulator |
| 20 | Butyl Isobutyl Phthalate | 36.94 | 0.09 | $C_{16}H_{22}O_4$ | 278 | 1924 $^{lit}$ | 17851-53-5 | 87 | ester | significant hypoglycemic effect potential for diabetes treatment |
| 21 | Hexadecanoic acid | 37.55 | 0.24 | $C_{16}H_{32}O_2$ | 256 | 1973 $^{lit}$ | 57-10-3 | 91 | carboxylic acid | antimycobacterial, anti-inflammatory |
| 22 | Linoleic acid | 44.91 | 0.07 | $C_{18}H_{32}O_2$ | 280 | 2104 $^{lit}$ | 60-33-3 | 91 | carboxylic acid | prostaglandin precursor essential fatty acid |
| 23 | Octadecanoic acid | 46.33 | 0.05 | $C_{18}H_{36}O_2$ | 284 | 2172 $^{lit}$ | 57-11-4 | 83 | carboxylic acid | pharmaceutical excipient |
| 24 | 2-(3-Methylbenzyl)-1-naphthoic acid | 51.76 | 0.12 | $C_{19}H_{16}O_2$ | 276 | N/A | 69653-22-1 | 83 | carboxylic acid | - |
| 25 | (2,5-diphenyl-1H-pyrrol-3-yl)-(2-naphthalenyl)methanone | 62.87 | 1.52 | $C_{27}H_{19}NO$ | 373 | N/A | 132058-99-2 | 72 | pyrrole | - |
| 26 | 6-phenyl-2-[(4,6,8-trimethyl-2-quinazolinyl)amino]-4-pyrimidinol | 65.84 | 5.03 | $C_{21}H_{19}N_5O$ | 357 | N/A | 2000502-50-2 | 91 | diazine | - |
| 27 | 3,3':5,3''-bis(dimethylene)-2,6-di(1',8'-naphthyrid-2'-yl)pyridine | 68.17 | 0.26 | $C_{25}H_{17}N_5$ | 387 | N/A | 96427-33-7 | 78 | pyridine | - |
| 28 | Mammea-E/BB | 71.12 | 1.37 | $C_{24}H_{30}O_7$ | 430 | N/A | 111321-12-1 | 91 | coumarin | cytotoxic |
| 29 | Mammea-B/AC | 74.16 | 2.37 | $C_{21}H_{26}O_5$ | 358 | N/A | 38537-84-7 | 83 | coumarin | - |
| 30 | 10,13-Di-t-butyl-14H-benzo [6,7]cyclohepta[1,2-b]naphtho[1,2-d]indole-14-one | 75.78 | 2.59 | $C_{29}H_{29}NO$ | 407 | N/A | 2000572-59-1 | 74 | indole | - |
| 31 | N,N'-bis[ethoxy(phenyl)phosphoryl]ethane-1,2-diamine | 78.73 | 1.12 | $C_{18}H_{26}N_2O_4P_2$ | 396 | N/A | 2000557-62-4 | 72 | amine | - |
| 32 | $\alpha$-Tocopherol | 81.44 | 0.40 | $C_{29}H_{50}O_2$ | 430 | 3130 $^{lit}$ | 59-02-9 | 97 | phenol | antioxidant, nutraceutical antiatherogenic, antiviral protein kinase C inhibitor anticoagulant, immunomodulator prevents cell membrane oxidative damage, anti-sterility properties pharmaceutical excipient |
| 33 | 1,4-Diphenyltriphenylene-2,3-diol | 87.68 | 1.67 | $C_{30}H_{20}O_2$ | 412 | N/A | 2000578-83-0 | 90 | alcohol | - |
| 34 | $\gamma$-Sitosterol | 88.62 | 0.86 | $C_{29}H_{50}O$ | 414 | N/A | 83-47-6 | 93 | steroid | plant metabolite |
| 35 | Mammea-A/AB | 90.12 | 9.62 | $C_{25}H_{26}O_5$ | 406 | N/A | 7058-70-0 | 92 | coumarin | against Gram-positive bacteria cytotoxic against human cancer cell lines, inhibitory of I$\kappa$B$\alpha$ kinase |
| 36 | Taraxasterol | 91.99 | 0.19 | $C_{30}H_{50}O$ | 426 | N/A | 1059-14-9 | 86 | triterpenoid | anti-inflammatory |

| | | | | | | | | | | |
|---|---|---|---|---|---|---|---|---|---|---|
| 37 | Friedelin | 100.25 | 0.97 | $C_{30}H_{50}O$ | 426 | 3510 [lit] | 559-74-0 | 95 | triterpenoid | non-narcotic analgesic antimicrobial, anti-inflammatory, anticonvulsant, antipyretic plant metabolite |
| | Identified Total Area % | | 40.46 | | | | | | | |

RT = retention time, M.F. = molecular formula, M.W. = molecular weight, LRI = linear retention index, lit = LRI obtained from literature, CAS No. = Chemical Abstracts Registry Number, N/A = not available

**Table S2**. GC-MS phytochemical profile for *Mammea americana* dichloromethane (MAD) leaf extract (full version).

| ID No. | Phytocompound | RT (min) | Area (%) | M.F. | M.W. | LRI [21] | CAS No. | Match Quality(%) | Classification | Biological Activity [22,23] |
|---|---|---|---|---|---|---|---|---|---|---|
| 1 | 6-Methyl-5-hepten-2-one | 4.45 | 0.07 | $C_8H_{14}O$ | 126 | 710 | 110-93-0 | 72 | ketone | insecticidal, alarm pheromone plant metabolite |
| 2 | trans-3-Hexenoic acid | 4.80 | 0.07 | $C_6H_{10}O_2$ | 114 | 714 | 1577-18-0 | 70 | carboxylic acid | plant metabolite |
| 3 | 2-Chlorocyclohexanone | 5.86 | 0.29 | $C_6H_9ClO$ | 132 | 726 | 822-87-7 | 90 | ketone | - |
| 4 | Dihydrodihydroxymaltol | 7.68 | 0.07 | $C_6H_8O_4$ | 144 | 1130 [lit] | 28564-83-2 | 90 | pyrone | strong antioxidant in glucose–histidine Maillard reaction products |
| 5 | Naphthalene,1,2-dihydro-1,5,8-trimethyl- | 11.45 | 0.02 | $C_{13}H_{16}$ | 172 | 1366 | 4506-36-9 | 91 | benzene derivative | - |
| 6 | Copaene | 12.02 | 0.17 | $C_{15}H_{24}$ | 204 | 1382 | 3856-25-5 | 99 | sesquiterpene | antibacterial |
| 7 | Isocaryophyllene | 12.83 | 0.04 | $C_{15}H_{24}$ | 204 | 1405 | 118-65-0 | 90 | sesquiterpene | fragrance ingredient |
| 8 | (-)-trans-Caryophyllene | 13.30 | 3.07 | $C_{15}H_{24}$ | 204 | 1419 | 87-44-5 | 99 | sesquiterpene | sedative, antibacterial, flavoring agent |
| 9 | β-Bisabolene | 13.56 | 0.08 | $C_{15}H_{24}$ | 204 | 1426 | 495-61-4 | 91 | sesquiterpene | plant metabolite, selective cytotoxicity |
| 10 | α-Humulene | 14.29 | 0.42 | $C_{15}H_{24}$ | 204 | 1447 | 6753-98-6 | 95 | sesquiterpene | anticarcinogenic, anti-inflammatory flavoring agent |
| 11 | trans-.beta.-Farnesene | 14.56 | 2.81 | $C_{15}H_{24}$ | 204 | 1455 | 502-60-3 | 97 | sesquiterpene | alarm pheromone |
| 12 | (3Z,6E)-alpha.-Farnesene | 15.90 | 0.44 | $C_{15}H_{24}$ | 204 | 1494 | 26560-14-5 | 90 | sesquiterpene | - |
| 13 | (E,E)-alpha-Farnesene | 16.52 | 2.05 | $C_{15}H_{24}$ | 204 | 1509 | 502-61-4 | 95 | sesquiterpene | codling moth attractant, flavoring agent |
| 14 | (+)-δ-Cadinene | 16.96 | 0.04 | $C_{15}H_{24}$ | 204 | 1520 | 483-76-1 | 94 | sesquiterpene | antibacterial, insecticidal, anticancer |
| 15 | beta-epoxide-Caryophyllene | 19.30 | 0.24 | $C_{15}H_{24}O$ | 220 | 1574 | 1139-30-6 | 93 | sesquiterpenoid | flavoring agent |
| 16 | Neophytadiene | 31.55 | 0.09 | $C_{20}H_{38}$ | 278 | 1838 [lit] | 504-96-1 | 95 | diterpene | anti-inflammatory, antimicrobial |

| | | | | | | | | | | |
|---|---|---|---|---|---|---|---|---|---|---|
| | | | | | | | | | | plant and algal metabolite |
| 17 | di-Isobutyl Phthalate | 32.65 | 0.12 | $C_{16}H_{22}O_4$ | 278 | 1877 [lit] | 84-69-5 | 78 | ester | teratogenic agent, PPAR modulator |
| 18 | Neophytadiene, Isomer III | 33.52 | 0.04 | $C_{20}H_{38}$ | 278 | 1883 [lit] | 504-96-1 | 93 | diterpene | anti-inflammatory, antimicrobial plant and algal metabolite |
| 19 | Dibutylphthalate | 36.99 | 0.07 | $C_{16}H_{22}O_4$ | 278 | 1907 [lit] | 84-74-2 | 86 | ester | insect repellent, glycosidase inhibitor |
| 20 | Hexadecanoic acid | 37.66 | 0.22 | $C_{16}H_{32}O_2$ | 256 | 1973 [lit] | 57-10-3 | 98 | carboxylic acid | antimycobacterial, anti-inflammatory |
| 21 | 3-Eicosene | 42.73 | 0.04 | $C_{20}H_{40}$ | 280 | N/A | 74685-33-9 | 72 | hydrocarbon | - |
| 22 | Linoleic acid | 45.00 | 0.07 | $C_{18}H_{32}O_2$ | 280 | 2104 [lit] | 60-33-3 | 74 | carboxylic acid | prostaglandin precursor essential fatty acid |
| 23 | Octadecanoic acid | 46.44 | 0.05 | $C_{18}H_{36}O_2$ | 284 | 2172 [lit] | 57-11-4 | 97 | carboxylic acid | pharmaceutical excipient |
| 24 | 1-Docosene | 59.48 | 0.06 | $C_{22}H_{44}$ | 308 | 2194 [lit] | 1599-67-3 | 78 | hydrocarbon | - |
| 25 | Cyclotetracosane | 67.00 | 0.49 | $C_{24}H_{48}$ | 336 | 2589 [lit] | 297-03-0 | 94 | hydrocarbon | - |
| 26 | Mammea-E/BB | 71.58 | 4.72 | $C_{24}H_{30}O_7$ | 430 | N/A | 111321-12-1 | 80 | coumarin | cytotoxic |
| 27 | Mammea-B/AB | 74.05 | 3.15 | $C_{22}H_{28}O_5$ | 372 | N/A | 111761-56-9 | 70 | coumarin | - |
| 28 | Mammea-B/BC | 74.42 | 2.30 | $C_{21}H_{26}O_5$ | 358 | N/A | 5085-54-1 | 83 | coumarin | cytotoxic |
| 29 | Herqueinone | 74.73 | 0.58 | $C_{20}H_{20}O_7$ | 372 | N/A | 26871-30-7 | 90 | phenalenone | - |
| 30 | 10,13-Di-t-butyl-14H-benzo [6,7]cyclohepta[1,2-b]naphtho[1,2-d]indole-14-one | 75.92 | 1.59 | $C_{29}H_{29}NO$ | 407 | N/A | 2000572-59-1 | 74 | indole | - |
| 31 | $\alpha$-Tocopherol | 81.57 | 0.49 | $C_{29}H_{50}O_2$ | 430 | 3130 [lit] | 59-02-9 | 97 | phenol | antioxidant, nutraceutical, antiatherogenic, antiviral protein kinase C inhibitor anticoagulant, immunomodulator prevents cell membrane oxidative damage, anti-sterility properties pharmaceutical excipient |
| 32 | Mammea-A/BD | 86.70 | 6.96 | $C_{24}H_{24}O_5$ | 392 | N/A | 16981-21-8 | 72 | coumarin | cytotoxic |
| 33 | Cholestane, ethanone derivative | 88.15 | 2.14 | $C_{29}H_{48}O$ | 412 | N/A | 2310-32-9 | 83 | steroid | - |
| 34 | β-Sitosterol | 88.91 | 0.95 | $C_{29}H_{50}O$ | 414 | 3187 [lit] | 83-46-5 | 70 | steroid | antihypercholesterolaemic, estrogenic, hypolipidemic, antibacterial, antifungal |
| 35 | Mammea-A/AB | 90.83 | 12.02 | $C_{25}H_{26}O_5$ | 406 | N/A | 7058-70-0 | 95 | coumarin | against Gram-positive bacteria cytotoxic against human cancer cell lines inhibitory of IκBα kinase |

| | | | | | | | | | | |
|---|---|---|---|---|---|---|---|---|---|---|
| 36 | 2-(1,3-Benzodioxol-5-yl)-3-methoxy-6-(pyridin-2-ylmethylsulfanyl)imidazo[1,2-b]pyridazine | 91.60 | 3.58 | $C_{20}H_{16}N_4O_3S$ | 392 | N/A | 2000552-75-6 | 91 | diazine | - |
| 37 | 1,3-diphenyl-4-[(phenylmethyl)amino]-5,6,7,8-tetrahydroquinolin-2-one | 92.04 | 1.67 | $C_{28}H_{26}N_2O$ | 406 | N/A | 83609-84-1 | 74 | quinolone | - |
| 38 | Friedelin | 100.79 | 1.41 | $C_{30}H_{50}O$ | 426 | 3510 lit | 559-74-0 | 97 | triterpenoid | non-narcotic analgesic, antimicrobial anti-inflammatory, anticonvulsant, antipyretic, plant metabolite |
| | Identified Total Area % | | 52.69 | | | | | | | |

RT = retention time, M.F. = molecular formula, M.W. = molecular weight, LRI = linear retention index, lit = LRI obtained from literature, CAS No. = Chemical Abstracts Registry Number, N/A = not available

**Table S3**. GC-MS phytochemical profile for *Mammea americana* methanolic (MAM) leaf extract (full version).

| ID No. | Phytocompound | RT (min) | Area (%) | M.F. | M.W. | LRI [21] | CAS No. | Match Quality(%) | Classification | Biological Activity [22,23] |
|---|---|---|---|---|---|---|---|---|---|---|
| 1 | 5-Methylfurfural | 3.95 | 0.06 | $C_6H_6O_2$ | 110 | 705 | 620-02-0 | 94 | aldehyde | flavoring agent |
| 2 | 2-Furoic acid methyl ester | 6.58 | 0.63 | $C_6H_6O_3$ | 126 | 980 lit | 611-13-2 | 80 | ester | flavoring agent |
| 3 | Dihydrodihydroxymaltol | 8.08 | 3.39 | $C_6H_8O_4$ | 144 | 1130 lit | 28564-83-2 | 91 | pyrone | strong antioxidant in glucose–histidine Maillard reaction products |
| 4 | Benzenecarboxylic acid | 8.47 | 0.27 | $C_7H_6O_2$ | 122 | 1170 lit | 65-85-0 | 93 | carboxylic acid | plant metabolite, antimicrobial antiseptic, expectorant, antifungal antipyretic, keratolytic, phytotoxic acaricide, antifouling |
| 5 | 4H-Pyran-4-one,3,5-dihydroxy-2-methyl- | 8.76 | 0.13 | $C_6H_6O_4$ | 142 | 1188 lit | 1073-96-7 | 93 | pyrone | - |
| 6 | 5-Hydroxymethyl-2-furaldehyde | 10.07 | 7.76 | $C_6H_6O_3$ | 126 | 1326 | 67-47-0 | 86 | furan | antibacterial |
| 7 | 2-Methoxy-4-vinyl-phenol | 10.66 | 0.20 | $C_9H_{10}O_2$ | 150 | 1343 | 7786-61-0 | 93 | phenol | plant metabolite, pheromone, antifungal |
| 8 | Copaene | 12.02 | 0.20 | $C_{15}H_{24}$ | 204 | 1382 | 3856-25-5 | 99 | sesquiterpene | antibacterial |
| 9 | (-)-trans-Caryophyllene | 13.28 | 3.41 | $C_{15}H_{24}$ | 204 | 1418 | 87-44-5 | 99 | sesquiterpene | sedative, antibacterial, flavoring agent |
| 10 | β-Sesquiphellandrene | 13.56 | 0.13 | $C_{15}H_{24}$ | 204 | 1426 | 20307-83-9 | 81 | sesquiterpene | plant metabolite |

| | | | | | | | | | | |
|---|---|---|---|---|---|---|---|---|---|---|
| 11 | $\alpha$-Humulene | 14.29 | 0.64 | $C_{15}H_{24}$ | 204 | 1447 | 6753-98-6 | 90 | sesquiterpene | anticarcinogenic, anti-inflammatory flavoring agent |
| 12 | trans-.beta.-Farnesene | 14.54 | 3.28 | $C_{15}H_{24}$ | 204 | 1455 | 502-60-3 | 98 | sesquiterpene | alarm pheromone |
| 13 | trans-$\alpha$-Bergamotene | 15.89 | 0.46 | $C_{15}H_{24}$ | 204 | 1493 | 13474-59-4 | 72 | sesquiterpene | - |
| 14 | (E,E)-alpha-Farnesene | 16.47 | 1.47 | $C_{15}H_{24}$ | 204 | 1508 | 502-61-4 | 97 | sesquiterpene | codling moth attractant, flavoring agent |
| 15 | β-Cadinene | 16.94 | 0.10 | $C_{15}H_{24}$ | 204 | 1519 | 523-47-7 | 93 | sesquiterpene | - |
| 16 | 3-Hydroxy-benzoic acid | 17.95 | 1.17 | $C_7H_6O_3$ | 138 | 1543 | 99-06-9 | 95 | carboxylic acid | plant metabolite |
| 17 | beta-epoxide-Caryophyllene | 19.31 | 0.31 | $C_{15}H_{24}O$ | 220 | 1574 | 1139-30-6 | 76 | sesquiterpenoid | flavoring ingredient |
| 18 | 4-Hydroxy-3-methoxybenzoic acid | 20.00 | 0.25 | $C_8H_8O_4$ | 168 | 1590 | 121-34-6 | 95 | carboxylic acid | plant metabolite, antifungal flavoring agent |
| 19 | Myristic acid | 28.20 | 0.11 | $C_{14}H_{28}O_2$ | 228 | 1765 [lit] | 544-63-8 | 72 | carboxylic acid | defoaming agent |
| 20 | 9H-Indeno[2,1-c]pyridin-9-one | 30.75 | 0.05 | $C_{12}H_7NO$ | 181 | N/A | 5061-91-6 | 72 | alkaloid | - |
| 21 | Neophytadiene | 31.54 | 0.14 | $C_{20}H_{38}$ | 278 | 1838 [lit] | 504-96-1 | 96 | diterpene | anti-inflammatory, antimicrobial plant and algal metabolite |
| 22 | Butyl Isobutyl Phthalate | 32.65 | 0.14 | $C_{16}H_{22}O_4$ | 278 | 1924 [lit] | 17851-53-5 | 78 | ester | significant hypoglycemic effect potential for diabetes treatment |
| 23 | Methyl hexadecanoate | 35.69 | 0.05 | $C_{17}H_{34}O_2$ | 270 | 1928 [lit] | 112-39-0 | 89 | ester | - |
| 24 | Dibutylphthalate | 36.99 | 0.08 | $C_{16}H_{22}O_4$ | 278 | 1970 [lit] | 84-74-2 | 90 | ester | insect repellent, glycosidase inhibitor |
| 25 | Hexadecanoic acid | 37.75 | 0.44 | $C_{16}H_{32}O_2$ | 256 | 1973 [lit] | 57-10-3 | 96 | carboxylic acid | antimycobacterial, anti-inflammatory |
| 26 | (Z)-9,17-Octadecadienal | 45.41 | 0.91 | $C_{18}H_{32}O$ | 264 | 1997 [lit] | 56554-35-9 | 81 | aldehyde | - |
| 27 | Octadecanoic acid | 46.48 | 0.14 | $C_{18}H_{36}O_2$ | 284 | 2172 [lit] | 57-11-4 | 89 | carboxylic acid | pharmaceutical excipient |
| 28 | Lycodoline | 65.85 | 3.13 | $C_{16}H_{25}NO_2$ | 263 | N/A | 6900-92-1 | 91 | alkaloid | anti-cholinesterase |
| 29 | 3-[(3-nitro-4-pyridinyl)amino]benzoic acid | 71.67 | 1.18 | $C_{12}H_9N_3O_4$ | 259 | N/A | 2000278-00-1 | 91 | carboxylic acid | - |
| 30 | Mammea-B/AB | 73.93 | 2.66 | $C_{22}H_{28}O_5$ | 372 | N/A | 111761-56-9 | 83 | coumarin | - |
| 31 | Mammea-B/AC | 74.24 | 1.83 | $C_{21}H_{26}O_5$ | 358 | N/A | 38537-84-7 | 83 | coumarin | - |
| 32 | Mammea-B/BA | 74.60 | 0.46 | $C_{22}H_{28}O_5$ | 372 | N/A | 521-38-0 | 83 | coumarin | significant cytotoxicity, insecticidal |
| 33 | 10,13-Di-t-butyl-14H-benzo[6,7]cyclohepta[1,2-b]naphtho[1,2-d]indole-14-one | 75.84 | 1.36 | $C_{29}H_{29}NO$ | 407 | N/A | 2000572-59-1 | 74 | indole | - |
| 34 | $\alpha$-Tocopherol | 81.49 | 0.38 | $C_{29}H_{50}O_2$ | 430 | 3130 [lit] | 59-02-9 | 94 | phenol | antioxidant, nutraceutical, antiatherogenic, antiviral protein kinase C inhibitor |

| | | | | | | | | | | |
|---|---|---|---|---|---|---|---|---|---|---|
| | | | | | | | | | | anticoagulant, immunomodulator prevents cell membrane oxidative damage, anti-sterility properties pharmaceutical excipient |
| 35 | Mesuol | 86.39 | 4.99 | $C_{24}H_{24}O_5$ | 392 | N/A | 16981-20-7 | 72 | coumarin | against Gram-positive bacteria cytotoxic against human cancer cell lines, inhibitor of IκB$\alpha$ kinase |
| 36 | 13,17-Diethyl-12,18-dimethyl-21,22-dioxaoxophlorin | 87.85 | 1.31 | $C_{26}H_{24}N_2O_3$ | 412 | N/A | 2000578-36-2 | 90 | porphyrin | - |
| 37 | $\gamma$-Sitosterol | 88.74 | 0.50 | $C_{29}H_{50}O$ | 414 | N/A | 83-47-6 | 99 | steroid | plant metabolite |
| 38 | Mammea-A/AB | 90.38 | 7.52 | $C_{25}H_{26}O_5$ | 406 | N/A | 7058-70-0 | 92 | coumarin | against Gram-positive bacteria cytotoxic against human cancer cell lines, inhibitor of IκB$\alpha$ kinase |
| 39 | Friedelin | 100.56 | 1.57 | $C_{30}H_{50}O$ | 426 | 3510 [lit] | 559-74-0 | 99 | triterpenoid | non-narcotic analgesic, antimicrobial anti-inflammatory, anticonvulsant antipyretic, plant metabolite |
| | Identified Total Area % | | 52.81 | | | | | | | |

RT = retention time, M.F. = molecular formula, M.W. = molecular weight, LRI = linear retention index, lit = LRI obtained from literature, CAS No. = Chemical Abstracts Registry Number, N/A = not available
